# Warming and Nitrogen Addition Increase Litter Decomposition in a Temperate Meadow Ecosystem

**DOI:** 10.1371/journal.pone.0116013

**Published:** 2015-03-16

**Authors:** Shiwei Gong, Rui Guo, Tao Zhang, Jixun Guo

**Affiliations:** 1 Institute of Grassland Sciences, Northeast Normal University, Key Laboratory for Vegetation Ecology, Ministry of Education, Changchun 130024, China; 2 Institute of Environment and Sustainable Development in Agriculture, Chinese Academy of Agricultural Sciences, Key Laboratory of Dryland Agriculture, Ministry of Agriculture, Beijing 100081, China; Helmholtz Centre for Environmental Research (UFZ), GERMANY

## Abstract

**Background:**

Litter decomposition greatly influences soil structure, nutrient content and carbon sequestration, but how litter decomposition is affected by climate change is still not well understood.

**Methodology/Principal Findings:**

A field experiment with increased temperature and nitrogen (N) addition was established in April 2007 to examine the effects of experimental warming, N addition and their interaction on litter decomposition in a temperate meadow steppe in northeastern China. Warming, N addition and warming plus N addition reduced the residual mass of *L*. *chinensis* litter by 3.78%, 7.51% and 4.53%, respectively, in 2008 and 2009, and by 4.73%, 24.08% and 16.1%, respectively, in 2010. Warming, N addition and warming plus N addition had no effect on the decomposition of *P*. *communis* litter in 2008 or 2009, but reduced the residual litter mass by 5.58%, 15.53% and 5.17%, respectively, in 2010. Warming and N addition reduced the cellulose percentage of *L*. *chinensis* and *P*. *communis*, specifically in 2010. The lignin percentage of *L*. *chinensis* and *P*. *communis* was reduced by warming but increased by N addition. The C, N and P contents of *L*. *chinensis* and *P*. *communis* litter increased with time. Warming and N addition reduced the C content and C:N ratios of *L*. *chinensis*and *P*. *communis* litter, but increased the N and P contents. Significant interactive effects of warming and N addition on litter decomposition were observed (P<0.01).

**Conclusion/Significance:**

The litter decomposition rate was highly correlated with soil temperature, soil water content and litter quality. Warming and N addition significantly impacted the litter decomposition rate in the Songnen meadow ecosystem, and the effects of warming and N addition on litter decomposition were also influenced by the quality of litter. These results highlight how climate change could alter grassland ecosystem carbon, nitrogen and phosphorus contents in soil by influencing litter decomposition.

## Introduction

Litter provides important energy and nutrient sources for microbial metabolism [1] and stores most of the belowground carbon in an ecosystem [2]. The factors associated with litter decomposition are primarily driven by micro- and macro-organismic activities (i.e., microbes, arthropods), litter quality (i.e., litter carbon (C), nitrogen (N), phosphorus (P), C:N ratios and lignin), climate (i.e., temperature and moisture), and the abundance of decomposers [3,4]. High quality litter (i.e., with low lignin content, high N and P contents and narrow C:N ratios) exhibits relatively fast decomposition rates [5,6,7]. Thus, litter decomposition rates are positively correlated with litter N and P contents and cellulose [8,9,10]. Therefore, any changes in litter quality and climate will affect litter decomposition. In addition, potential changes in decomposition rates and associated C loss and N and P release and/or immobilization from litter under global climate change will influence plant productivity and global C, N and P cycling [3,11].

It is predicted that global mean surface temperatures will rise by 1.1–6.4°C [12]. As a consequence, climate warming may directly alter soil temperature and moisture and the activity of soil organisms in ecosystems [3]. Nadelhoffer et al. reported that increased temperature enhances the rate of litter decomposition by stimulating microbial activity [13]. However, other studies revealed the opposite result, where the increase in temperature hinders the rate of decomposition because of moisture decline [14], as well as decreasing plant litter quality [15] and altering plant species composition of different litter qualities [14]. These changes in litter quality and species composition under warming have an indirect impact on litter decomposition [11]. Though the influence of climate warming on litter production, decomposition, and quality has been examined [16–18], the effects of warming on litter decomposition to C, N and P dynamics in litter pools at the ecosystem scale remain unclear.

Nitrogen deposition caused by anthropogenic activities is currently 30% greater than deposition from natural terrestrial inputs and is substantially greater than it was a hundred years ago [19]. This increase in N inputs is expected to affect ecosystem processes such as plant growth, plant species diversity, biogeochemical cycles, and net ecosystem C-accumulation [20], specifically in temperate terrestrial ecosystems where N deposition is limited. Though the effects of N added to soil through litter decomposition are inconsistent (i.e., positive [21–24], negative [9,25], and neutral effects [5,6] have been reported), N deposition still plays a key role in litter decomposition dynamics. Further, N addition can increase inorganic N availability and decrease litter C:N ratios [17,25] due to more rapid litter decomposition [21]. Currently, the effects of N deposition on litter decomposition remain controversial.

Numerous studies have focused on the individual effects of N addition and warming on litter quality; however, little attention has been paid to the interactive effects of warming and N addition on litter decomposition. Concurrent changes in global temperature and N addition may have potentially interactive effects on litter decomposition. Songnen Grassland lies in the eastern edge of the Eurasian grassland biome, which is the most typical and largest meadow steppe in China. The average temperature of the Songnen meadow steppe has risen 2°C in the last two decades [26], and average atmospheric N deposition is approximately 10.5 g m^-2^ a^-1^ [27]. To examine the influence of experimental warming and N addition on litter decomposition, we conducted a 3-year artificial warming and N addition experiment in the Songnen meadow steppe in northeast China. In this study, we addressed these questions: (1) to what extent do warming and N addition affect litter decomposition? and (2) are there interactive effects between warming and N addition on litter decomposition?

## Materials and Methods

### Study Site

The study was conducted in the Songnen Grassland at the Grassland Ecosystem Field Station of the Northeast Normal University in northeast China (123°44′ E and 144°40′ N). The long-term mean annual temperature is 6.4°C with a frost-free period of 141 days. The mean annual rainfall is 470 mm, which occurs between June and August. The annual potential evapotranspiration is 2–3 times higher than the annual rainfall [28]. The growing season is limited from late April to early October. The vegetation is dominated by the perennial grass *Leymus chinensis* (Trin.) Tzvel. and *Phragmites communis*, accompanying vegetation are *Kalimeris integrifolia* Turcz. Ex DC., *Carex duriuscula* C. A. Mey. and *Rhizoma phragmitis*. The height of the plant communities is 60 cm, and total community cover exceeds 80%. The primary soil type is Chernozem. The total soil N, organic C and pH, measured at a depth of 0 to 25 cm, are 19.6 ± 1.32 g kg^-1^, 29.39 ± 2.96 g kg^-1^, and 8.14 ± 0.2, respectively. The soluble salt content of the soil is high; the main cation is Na^+^, and the main anion is HCO_3_^-^[28].

### Experimental Design

The experiment was designed as a randomized complete block with warming and N addition as fixed factors. Each factor had two levels. There were four treatments: control (C), warming (W), N addition (N), and warming plus N addition (WN), with six replicates of each treatment. The size of each plot was 2 × 3 m. The warming plots were heated continuously using infrared radiators (Kalglo Electronics Inc. Bethlehem, PA, MSR-2420, USA) suspended at a height of 2.25 m over the center of each plot. In each control and N addition plot, a ‘dummy’ heater with the same shape and size was installed to simulate the shading effects of the infrared radiator. The heaters were set at a radiation output of approximately 1700 W.

He et al. estimated that airborne N up to 80–90 g m^-2^ yr^-1^ and higher N deposition would occur in the future owing to land-use change and anthropogenic activities [29]. In addition, Bai et al. estimated that the community saturation rate of N deposition was approximately 10.5 g m^-2^ yr^-1^ for a temperate grassland ecosystem [27]. Thus, in the N addition treatment plots in the current study, a pulse of aqueous ammonium nitrate (10 g m^-2^ yr^-1^) was added on the first day of May each year. The same amount of water (equivalent to ~ 2 mm of rainfall) was applied to N addition and ambient N plots (i.e., without N addition).

### Soil Temperature and Water Content Measurements

Soil temperature and water content were measured using an ECH_2_O Dielectric Aquameter (EM50/R Decagon Ltd, Pullman, WA, USA). In each subplot, soil temperature and water content (0–15 cm) were measured daily between 08:00 and 09:00 A.M. in late May, mid-June, mid-July, early August, mid-September and mid-October in 2008, 2009 and 2010.

### Litter Decomposition

In September 2007, we collected *Leymus chinensis* and *Phragmites communis* litter from areas outside the experimental blocks. The litter had recently been dropped on the ground surface and was dead but still connected to living plants. The litter samples were stored under ventilated conditions in the laboratory prior to decomposition experiments. In April 2008, the samples were air-dried in a ventilation oven to constant weight and cut into 10 cm long segments. The litter was placed in 25 × 15 cm bags constructed using 1 mm mesh nylon wire, and each litterbag contained 10 g of litter per species. The litterbags were fixed on the ground surface of the appropriate plots using metal pins to prevent movement from wind. The initial chemical composition of *L*. *chinensis* and *P*. *communis* differed (Table 1). *L*. *chinensis* had 55.4% higher N content, 10.3% higher P content, 18.5% lower C content, 47.5% lower C:N ratio, 11.3% lower cellulose and 24.2% lower lignin content compared with *P*. *communis*.

**Table 1:** Initial litter chemistry component of *L*. *chinensis* and *P*. *communis*

Species	C (mg/g)	N (mg/g)	P (mg/g)	C:N	Cellulose (%)	Lignin (%)
*L*. *chinensis*	321.4±6.0b	6.272±0.3a	0.86±0.02a	51.24±3b	36.58±2b	6.22±0.2b
*P*. *communis*	394.12±7.2a	4.037±0.2b	0.78±0.01b	97.63±7a	41.24±5a	8.21±0.3a

C = total C; N = total N; P = total P. Values presented are the means±S.E. (n = 6). Statistically significant differences (*P* < 0.05) between treatments are indicated by different lowercase letters.

The litterbags were retrieved in October of 2008, 2009 and 2010 and transported to the laboratory for further analyses. Living plants and plant tissues were removed, and soil particles were carefully wiped off. Samples were dried for 48 hours at 65°C and then weighed.

### Chemical Analysis

The dried samples were ground and passed through a 1 mm mesh. Total C and N contents of the samples were determined using the dichromate oxidation [30] and Kjeldahl methods [31]. Total P was first digested in sulfuric acid and subsequently quantified using an ICP Elemental Analyzer (Bruker Analysis Instrument Ltd., Karlsruhe, BW Germany). Specifically, 10 ml of sulfuric acid and 5 ml of perchloric acid were added to 0.7 g of soil and boiled for 1 hour at 420°C. Then, the solution was filtered, made up to a constant volume, and quantified using an ICP Elemental Analyzer. The cellulose and lignin contents were analyzed using the acid-detergent fiber method [32]. In addition, six litter samples from each plant species were analyzed for total C, N, P, cellulose and lignin to determine the initial litter chemistry.

### Statistical Analysis

The dry residual mass of the litter (*R*) during decomposition was calculated as follows:

R(%)=Mt/M0×100%

where *M0* is the initial dry mass of the litter before decomposition, *Mt* is the dry residual mass of litter in the litterbag after a specific time period (*t*) of decomposition.

A one-way ANOVA and repeated measures ANOVAs were used to assess the temporal (inter- or intra-annual) variation and effects of warming and N addition on soil temperature, soil water content, litter C, N, P, cellulose and lignin contents. Warming, N addition, and their interaction were treated as between-subject factors. Linear regression analyses were used to determine the relationships between litter decomposition and soil temperature, soil water content, litter C, N, P, cellulose and lignin contents. Post-hoc Tukey’s tests were performed to compare treatment differences. Statistical analyses were conducted using SPSS 16.0 software (SPSS Institute Inc., Chicago, IL, USA). Data are reported as the mean±SE.

## Results

### Soil Temperature and Water Content

**Fig 1:**
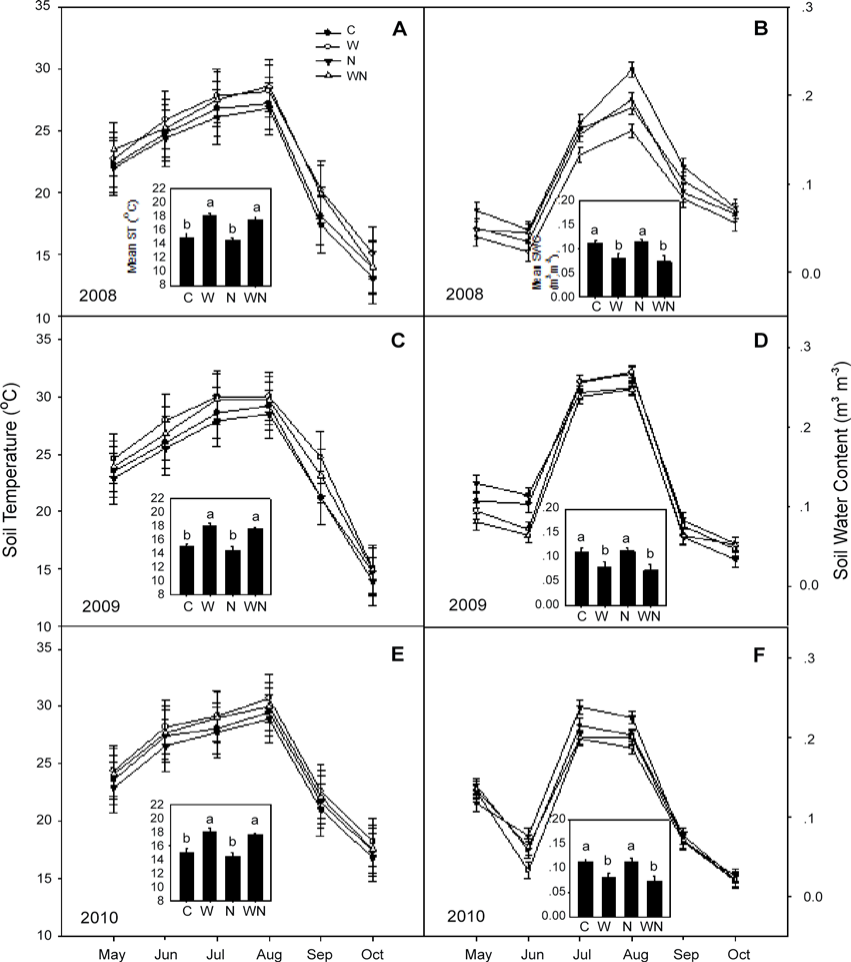
Effects of experimental warming and N addition on soil temperature and soil volumetric water content at a depth of 15 cm over the period 2008 to 2010. C: Control; W: Warming; N: N addition; WN: Warming plus N addition. Vertical bars indicate the standard error of the mean (n = 6). Different lowercase letters indicate significant differences (*P* < 0.05) between treatments.

Soil temperature displayed a seasonal response to treatments over the three growing seasons. Within a single growing season, the soil temperature (measured at a depth of 0–15 cm) exhibited a unimodal peak in August (Fig. 1A, C, E). In general, warming increased the soil temperature (*P*<0.05) by an average of 1.1°C, but N addition did not affect soil temperature. Warming in combination with N addition significantly increased soil temperature (*P*<0.05), but there was no difference between this combined treatment and warming alone.

Similar to the soil temperature response, the soil water content displayed a seasonal trend, with peak values in July and August (Fig. 1B, D, F). Warming significantly reduced the soil water content (*P*<0.05), whereas the addition of N alone had no effect compared with the control. The combination of warming and N addition reduced the soil water content (*P*<0.05); however, the difference between this combined treatment and warming alone was not significant.

### Litter Decomposition

**Fig 2:**
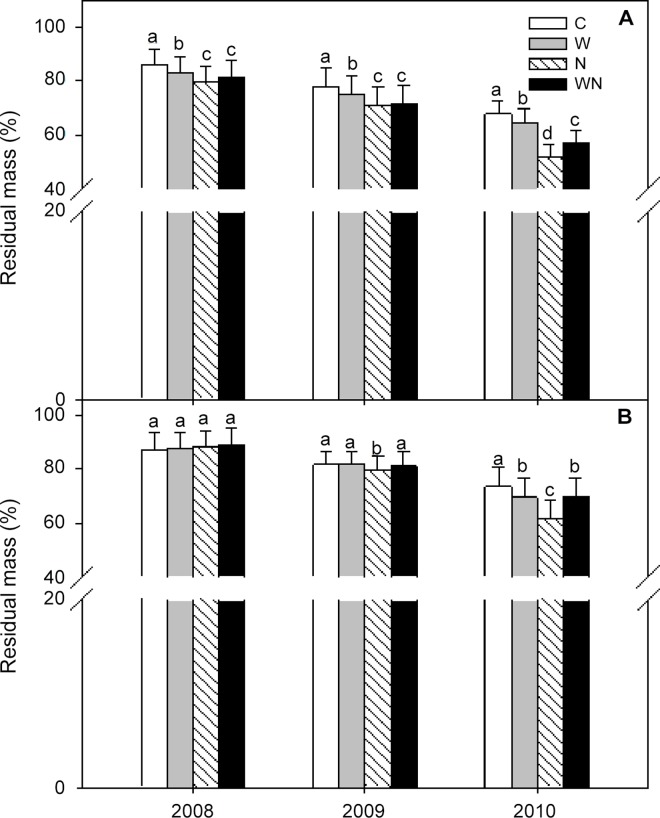
Effects of warming and N addition on the litter decomposition of *L*. *chinensis* (A) and *P*. *communis* (B) from 2008 to 2010. C: Control; W: Warming; N: N addition; WN: Warming plus N addition. Vertical bars represent the standard error of the mean (n = 6). Different lowercase letters indicate significant differences (*P* < 0.05) between treatments.

*L*. *chinensis* litter decomposed faster than *P*. *communis* litter (Fig. 2). After 3 years, litter loss was 21.8% higher (*P*<0.05) in *L*. *chinensis* compared with *P*. *communis* in the control plots.

Warming, N addition and warming plus N addition reduced the residual mass of *L*. *chinensis* litter by 3.78%, 7.51% and 4.53% (all *P* < 0.05), respectively, in 2008 and 2009, and by 4.73%, 24.08% and 16.1% (all *P* < 0.05), respectively, in 2010 (Fig. 2A). Warming, N addition and warming plus N addition had no effect on the decomposition of *P*. *communis* litter in 2008 or 2009, but reduced the residual litter mass by 5.58% (*P* < 0.05), 15.53% (*P*< 0.01) and 5.17% (*P* < 0.05), respectively, in 2010 (Fig. 2B). Therefore, the effect of N addition on litter decomposition was greater than the effect of warming, and warming, N addition and warming plus N addition had different effects on *L*. *chinensis* and *P*. *communis* litter. The residual mass of *L*. *chinensis* and *P*. *communis* litter was significantly (*P* < 0.001) affected by warming, N addition and the interaction between warming and N addition (Table 2).

**Table 2:** Results (*F*-value) of two-way factorial ANOVA on the effects of specie (S), warming (W), nitrogen addition (N) and their interaction on *P*-values from mixed statistics on the residual mass of litter, litter total carbon (C), total N (N), total P (P), cellulose and lignin.

	Residual mass	C	N	P	C:N	Cellulose	Lignin
S	117.312***	147.449***	27.618***	14.14*	18.361**	90.156***	12.145**
W	97.491***	100.216***	12.138*	13.701*	39.259**	111.874***	21.718**
N	268.84***	114.404***	9.047*	32.996**	31.649**	160.537***	74.849***
W×N	64.83***	223.229***	12.256*	0.875	68.969***	61.458***	13.651*
S×W	7.81*	20.346**	5.507	3.715	16.215**	4.433	15.889**
S×N	6.231	25.913**	16.024**	9.83*	7.301*	15.447**	47.898***
S×W×N	4.908	63.328***	7.215*	2.337	10.014*	3.728	10.356*

* denotes significant difference at *P* < 0.05;

** denotes significant difference at *P* < 0.01;

*** denotes significant difference at *P* < 0.001.

### Litter quality

**Fig 3:**
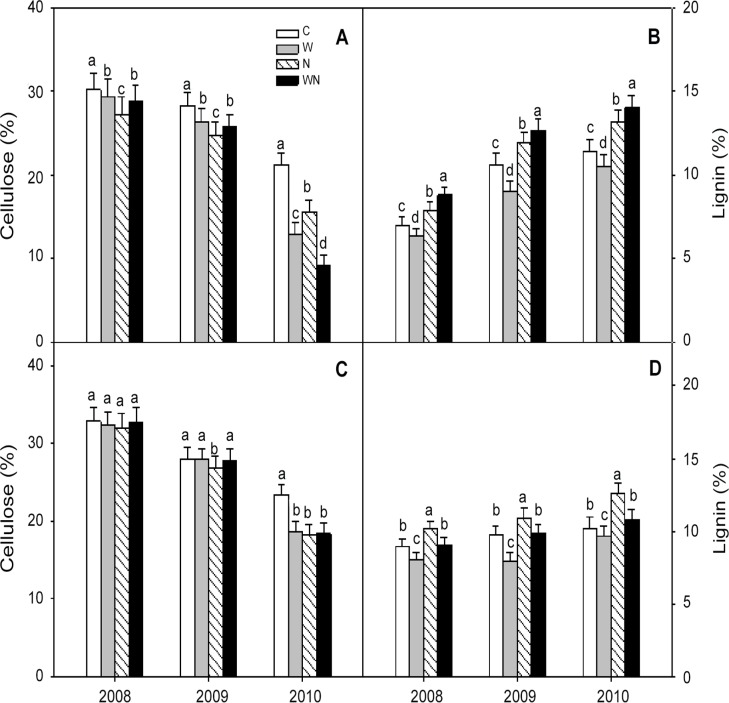
Effects of warming and N addition on cellulose and lignin contents of *L*. *chinensis* (A and B) and *P*. *communis* (C and D) from 2008 to 2010. C: Control; W: Warming; N: N addition; WN: Warming plus N addition. Vertical bars represent the standard error of the mean (n = 6). Different lowercase letters indicate significant differences (*P* < 0.05) between treatments.

After 3 years, the cellulose contents of *L*. *chinensis* and *P*. *communis* in the control plots decreased by 29.51% and 28.48% (all *P* < 0.05), respectively, and the lignin contents increased by 60.03% and 14.38% (all *P* < 0.05), respectively (Fig. 3).

Warming, N addition and warming plus N addition reduced the cellulose content of *L*. *chinensis* litter by 2.54%, 9.61% and 4.66% (all *P* < 0.05), respectively, in 2008 and 2009, and by 39.28%, 26.57% and 56.93% (all *P* < 0.05), respectively, in 2010 (Fig. 3A). Warming, N addition and warming plus N addition had no effect on the cellulose content of *P*. *communis* litter in 2008 and 2009 but reduced the cellulose content by 20.31% (*P* < 0.05), 22.16% (*P*< 0.01) and 21.56% (*P* < 0.05), respectively, in 2010 (Fig. 3C). Therefore, N addition had a greater effect on the cellulose decomposition of *L*. *chinensis* litter compared with warming in 2008 and 2009, but the effect of warming was greater than that of N addition in 2010.

During the 3 years of decomposition, warming reduced the lignin content of *L*. *chinensis* and *P*. *communis* litter by 9.88% and 9.41% (all *P* < 0.05), respectively (Fig. 3B, D), N addition increased the lignin contents by 12.52% and 14.15% (all *P* < 0.05), respectively (Fig. 3B, D), and warming plus N addition increased the lignin content of *L*. *chinensis* litter by 25.63% (*P* < 0.05), but had no effect on the lignin content of *P*. *communis* litter.

**Fig 4:**
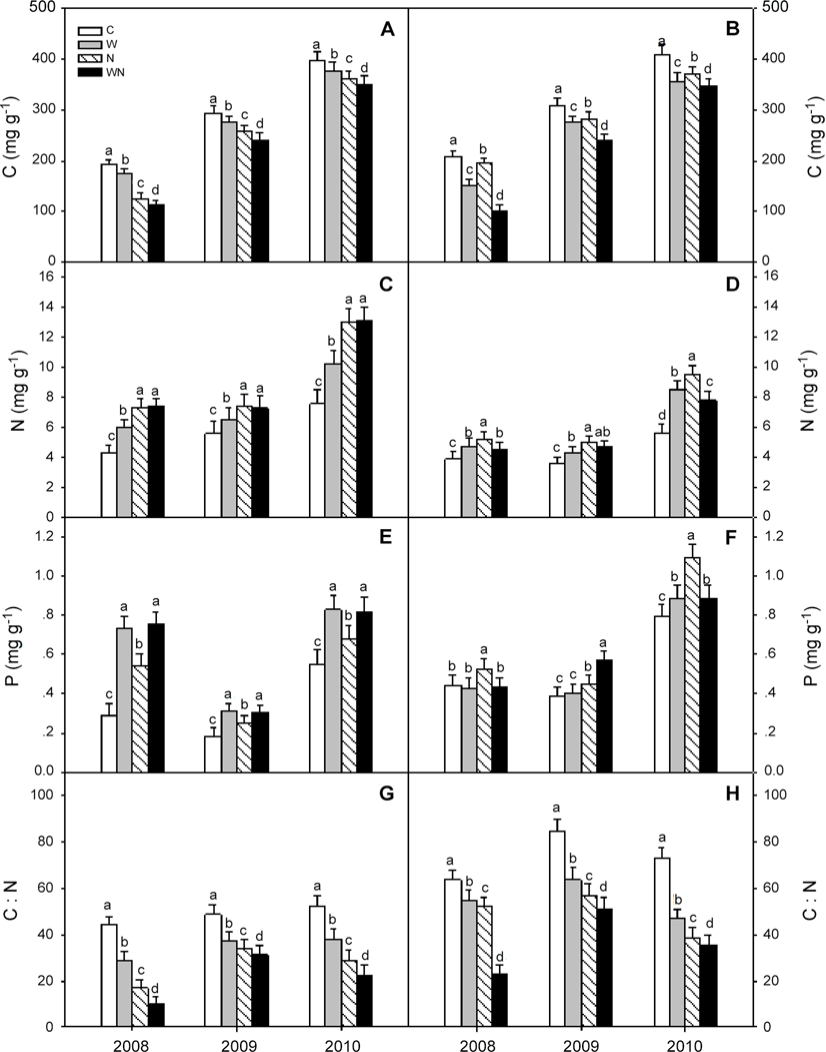
Effects of warming and N addition on litter quality of *L*. *chinensis* (A, C, E and G) and *P*. *communis* (B, D, F and H) from 2008 to 2010. C: Control; W: Warming; N: N addition; WN: Warming plus N addition. Vertical bars represent the standard error of the mean (n = 6). Different lowercase letters indicate significant differences (*P* < 0.05) between treatments.

The C and N contents of *L*. *chinensis* and *P*. *communis* litter increased over time in the control plots (Fig. 4). During the 3 years of decomposition, warming, N addition and warming plus N addition reduced the C contents and C:N ratios of *L*. *chinensis* and *P*. *communis* litter (*P* < 0.001) (Fig. 4 A, B, G, H), but increased the N and P contents of *L*. *chinensis* (Fig. 4C, E) and N content of *P*. *communis* litter (Fig. 4D) (*P* < 0.05). Warming increased the P content of *P*. *communis* litter in 2010 (*P* < 0.05), but had no effect in 2008 or 2009, and N addition increased the P content of *P*. *communis* litter (*P* < 0.05) (Fig. 4F).

The C, N and P content, C:N ratio, and the cellulose and lignin contents of the *L*. *chinensis* and *P*. *communis* litter were significantly (*P* < 0.001) affected by warming, N addition and the interaction between warming and N addition (Table 2).

Correlation of litter decomposition with soil microclimate and litter quality

Correlation analysis showed that the residual mass of *L*. *chinensis* and *P*. *communis* litter was positively correlated with soil temperature, water content, cellulose and N, P content (*P* < 0.05), but negatively correlated with the C:N ratio (*P* < 0.01) and lignin content (*P* < 0.05). The correlation coefficients ranged from 0.286 to 0.984 (Table 3).

**Table 3:** Correlations between the residual mass of litter and litter quality, soil temperature, and soil water content.

Residual mass	ST	SWC	C	N	P	C:N	Cellulose	Lignin
*L*. *chinensis*	0.823**	0.920**	0.929*	0.944**	0.382*	-0.936**	0.984*	-0.884***
*P*. *communis*	0.642*	0.565*	0.286*	0.676**	0.983***	-0.550**	0.960*	-0.722*

C = total C; N = total N; P = total P; ST = soil temperature, SWC = soil water content.

*denotes significant difference at *P* < 0.05;

** denotes significant difference at *P* < 0.01;

*** denotes significant difference at *P* < 0.001.

## Discussion

### Effects of Warming on Litter Decomposition

A stimulatory effect of warming on decomposition rates has been found in other studies [10,14,33,34,35]. In our experiment, warming increased litter decomposition rates although the effect was weak. An increase in temperature can increase litter decomposition rates directly by affecting the soil microclimate and stimulating microbial activity [13], or indirectly by improving litter quality [11]. After 3 years of warming, most of the initial litter chemistry parameters had changed considerably, e.g., warming increased N and P contents, and reduced C contents, C:N ratios, cellulose and lignin contents. These results indicate that warming improved litter quality, resulting in increased litter decomposition rates. And warming increased litter decomposition rates by changing grassland community structure and plant tissue quality have also been reported [36,37]. These studies found that warming increased the annual aboveground biomass, increased the output of litter, and warming increased the N, P contents of plant tissue, improved the quality of plant tissue, which benefited decomposition of litter. Warming had a greater effect on the decomposition of *L*. *chinensis* litter compared with *P*. *communis* litter. The decomposition rate and temperature sensitivity of high quality litter quality is higher than low litter quality [11,34,35]. In the current study, the litter quality of *L*. *chinensis* was higher than *P*. *communis*.

Nitrogen is frequently immobilized during litter decomposition [38], and in the current study, warming enhanced this phenomenon and consequently increased N contents. An increase in C leaching and a reduction in the C:N ratio has been observed in response to warming [39]. Warming stimulates the litter decomposition rate, which reduces cellulose and lignin contents, and subsequently decreases C storage [14,17].

The negative correlations observed between the litter decomposition rates and C:N ratios and lignin agree with the results of previous studies [14,15,40]. *L*. *chinensis* litter had higher N and P contents, but lower C contents, C:N ratios, cellulose and lignin contents than *P*. *communis*. Therefore, *L*. *chinensis* litter decomposed faster than *P*. *communis*. Though elevated soil temperatures under climate warming may accelerate litter decomposition, warming also decreases soil moisture, which suppresses soil microbial activity [14,41]. It is possible that the more rapid decomposition associated with improved litter quality in the warming plots may have counteracted slow decomposition rates caused by decreased soil moisture and soil microbial activity.

Warming had little effect on *P*. *communis* litter decomposition in 2008 and 2009, possibly because losses in the early phase of decomposition consist of easily degraded soluble compounds and celluloses [42], after which *P*. *communis* likely had higher lignin and C contents.

### Effects of N Addition on Litter Decomposition

The mass loss of litter was accelerated by N addition, indicating that an increase in N availability accelerated litter decomposition, which is consistent with previous results [21,22,43]. However, other studies found that N addition had no effect [6], or even depressive effects [8,25,44] on litter decomposition. These findings can be attributed to differences in the amount of N added, fertilizer types and species [6,45].

After 3 years of N addition, most of the initial litter chemistry parameters had changed considerably. N addition increased N, P and lignin contents, but decreased C, cellulose contents and C:N ratios. These results indicate that N addition improved litter quality, resulting in increased litter decomposition rates. Similar results have been observed in other studies [17,46]. Nitrogen addition had a greater effect on the decomposition of *L*. *chinensis* litter compared with *P*. *communis* litter. Specifically, N addition reduced the residual mass of *L*. *chinensis* in all 3 years, whereas this treatment did not affect the decomposition of *P*. *communis* litter in 2008 and 2009, but reduced the residual mass of *P*. *communis* litter in 2010. *L*. *chinensis* litter with a higher N content and lower C content and C:N ratio decomposed faster than low-quality *P*. *communis* litter [47]. In addition, *P*. *communis* litter had a high lignin content, and cellulose and compounds that are easily degraded are decomposed during the early phase of decomposition [42]. Further, N addition may indirectly stimulate litter decomposition rates by increasing the N content of the litter. The balance between N and P contents is important because several nutrient contents in litter are frequently correlated, and nutrient balance is necessary for microbial growth [7,40]. N addition can inhibit the decomposition of lignin by suppressing the synthesis of ligninolytic enzymes or promoting the formation of additional recalcitrant compounds through interactions between N and lignin breakdown products. In addition, added N can promote the decomposition of cellulose by increasing the activity of cellulose-degrading enzymes because shifts in enzyme activity can influence litter decomposition [5,7,8,25]. It is generally believed that N addition accelerates the decomposition of rapidly decomposing substrates (i.e., cellulose) but slows the breakdown of slowly decomposing materials (i.e., lignin) [45,48]. Dias et al. accounted for the integrated effects of N enrichment on litter decomposability taking into consideration the N-driven changes in the whole plant community (changes in plant species composition and litter quality), perhaps by altering the competitive interactions between species [49]. Maybe N additions reduced the abundance of *P*. *communis* and benefited *L*. *chinensis*, which resulted in greater biomass stimulation of *L*. *chinensis*, greater plant tissue quality and greater quality of litter inputs [49,50]. Therefore, N addition had a greater effect on *L*. *chinensis* litter decomposition compared with *P*. *communis*.

### Interactive Effects between Warming and N Addition on Litter Decomposition

Litter decomposition rates were stimulated by the interactive effects of warming and N addition. Warming and N addition may increase litter decomposition rates directly by stimulating microbial activity (data not shown) or indirectly by improving litter quality. The interactive effect between warming and N addition reduced C contents and C:N ratios, but increased N and P contents and therefore improved litter quality. High-quality litter exhibited relatively faster decomposition, which is consistent with earlier studies [7]. There was a greater interactive effect between warming and N addition on *L*. *chinensis* litter decomposition compared with *P*. *communis*. This result is because the interactive effect resulted in higher N and P and lower cellulose contents in *L*. *chinensis* litter compared with *P*. *communis*, resulting in litter that was more easily decomposed.

## Conclusions

Changes in abiotic (soil temperature and moisture) environments and litter quality play a vital role in litter decomposition in temperate meadow grasslands. As rates of litter decomposition increase with warming and N addition in temperate meadow grasslands, the turnover times of C, N and P from the litter to soil will decrease. This has the potential to increase the storage of C, N and P in the soil, and might alter the timing and availability of these nutrients for plant growth.

Our findings suggest that plant species with different litter qualities should be considered when modeling C and N cycles and nutrient dynamics in grassland ecosystems. In addition, *L*. *chinensis* is expected to contribute more than *P*. *communis* to C and N cycling and nutrient dynamics in the semi-arid Songnen grasslands. Moreover, our results further the current understanding of litter decomposition in response to multiple global change drivers in temperate grassland ecosystems.
